# Correct response negativity may reflect subjective value of reaction time under regulatory fit in a speed‐rewarded task

**DOI:** 10.1111/psyp.13856

**Published:** 2021-06-06

**Authors:** Benjamin T. Files, Kimberly A. Pollard, Ashley H. Oiknine, Peter Khooshabeh, Antony D. Passaro

**Affiliations:** ^1^ US Army Combat Capabilities Development Command Army Research Laboratory Los Angeles CA USA; ^2^ DCS Corporation Los Angeles CA USA; ^3^ Department of Psychological and Brain Sciences University of California Santa Barbara CA USA

**Keywords:** EEG, ERPs, Error Processing, Individual Differences, Motivation

## Abstract

Error‐related negativity (ERN), an electroencephalogram (EEG) component following an erroneous response, has been associated with the subjective motivational relevance of error commission. A smaller EEG event, the correct response negativity (CRN), occurs after a correct response. It is unclear why correct behavior evokes a neural response similar to error commission. CRN might reflect suboptimal performance: in tasks where speed is motivationally relevant (i.e., incentivized), a correct but slow response may be experienced as a minor error. The literature is mixed on the relationship between CRN and response time (RT), possibly due to different motivational structures, tasks, or individual traits. We examined ERN and CRN in a go/no‐go task where correctness and speed were encouraged using a points‐based feedback system. A key individual trait, regulatory focus, describes a person's tendency to seek gains (promotion focus) and avoid losses (prevention focus). Trait regulatory focus was measured, and participants were randomly assigned to one of three conditions: points gain, points loss, and informative‐only feedback. Participants committed too few errors to reliably model ERN effects. CRN amplitude related to RT in all feedback conditions, with slower responses having larger CRN. Participants with stronger promotion focus had a more exaggerated RT/CRN relationship in the point gain condition, suggesting that regulatory fit influences the motivational relevance of speed and thus the negative subjective experience and CRN for slower responses. These findings are consistent with the claim that CRN reflects RT when RT is motivationally relevant and that the CRN/RT relationship reflects the degree of subjective motivational relevance.

## Abstract 2

Our findings show that larger correct response negativities are associated with slower responses in a task with explicit response time feedback. Using regulatory fit theory, we further show that the correct response negativity reflects subjective motivational value from regulatory fit between promotion orientation strength and feedback framed as point gains. These findings suggest that correct response negativity reflects response time when response time is motivationally relevant.

**Highlights**

- The correct response negativity is a response‐related component of the EEG arising when a correct response is issued.
- We show that in a task with explicit feedback about response time, the correct response negativity is larger with slower responses.
- We also show that this relationship between response time and negativity is affected by individual regulatory focus and the framing of response time feedback.
- These findings are consistent with the view that the correct response negativity reflects the subjective and objective value of the speed of a response.

## 1 INTRODUCTION

The error‐related negativity (ERN; Gehring et al., 1993), also known as the error negativity (Ne; Falkenstein et al., 1990, 1991), and the related correct response negativity (CRN) are components of the electroencephalogram (EEG) that arise immediately following an overt response, typically peaking within 100 ms of a response (Gehring et al., 2011). When the response is in error, the subjective value or affective and motivational significance of the error is reflected in the amplitude of the ERN relative to baseline (Boksem et al., 2006; Hajcak, 2012; Hajcak et al., 2005; Legault & Inzlicht, 2013; Segalowitz & Dywan, 2009; Weinberg et al., 2012) with a more negative ERN associated with more motivationally significant errors. When the response is correct, a CRN is observed (Vidal et al., 2000). The CRN is less well‐studied, but its amplitude appears to reflect subjective uncertainty in the response correctness (Scheffers & Coles, 2000), response strategy evaluation (Bartholow et al., 2005), attentional control or effortful vigilance (Matsuhashi et al., 2021; van Noordt et al., 2015, 2016), and/or motivation to respond correctly (Imhof & Rüsseler, 2019), with a larger (more negative) CRN associated with more uncertainty, suboptimal strategy, greater attention or vigilance, and higher motivation, respectively. The CRN appears to have many properties in common with the ERN, and some evidence indicates that they share a common neural generator in the anterior cingulate (Roger et al., 2010); however, others have pointed out that approaches showing a common source use methods that are likely to obscure source differences (Endrass et al., 2012), and other methods point to the ERN and CRN having non‐identical neural generators (Endrass et al., 2012; Vocat et al., 2008), although there is general agreement that both ERN and CRN have a common peak over fronto‐central electrode sites.

In some cases, the CRN amplitude is observed to relate to response time, such that slower responses are associated with a larger CRN (Luu et al., 2000). The CRN is pronounced when associated with a correct response falling after an explicit response deadline (Heldmann et al., 2008), so a relation between CRN amplitude and response time might be due to a deadline or to general pressure to respond quickly (Coles et al., 2001), although this relationship might rely on the presence of external pressure to respond quickly (Vidal et al., 2003). This relationship could also arise as a result of partial error commission (Masaki & Segalowitz, 2004; Matsuhashi et al., 2021), wherein a participant begins to make an error, represses that initial response, and then makes the correct response. This process would lead to both a slower response time and a larger CRN. However, other work has shown a larger CRN associated with faster responding (Valt & Stürmer, 2017). The apparent inconsistency in the relationship between CRN amplitude and response time could be due to methodological differences. For example, stimulus‐evoked activity could mask differences in CRN amplitude for responses at different times relative to stimulus onset, and different baseline subtraction methods could also impact results. Moreover, under the hypothesis that CRN, like the ERN, reflects the subjective value of a response, differences in task structure, instructions, and feedback could also mediate the relationship between CRN and response time. Specifically, when fast responding is of high motivational relevance, we expect CRN amplitude to be positively associated with response time (RT), such that longer RTs are associated with more negative CRNs. We furthermore expect that increased motivational regimes (resulting from individual traits and a match between individual traits and reward structures) should exaggerate this relationship between CRN and RT.

In this report, we explore the relationship between the CRN amplitude, RT, and dispositional factors that are expected to modulate the subjective importance of responses through *regulatory fit* (Higgins, 1998, 2000; Maddox & Markman, 2010; Shah et al., 1998). Regulatory fit builds on regulatory focus theory (Higgins et al., 2001), which posits two general self‐regulatory foci: *promotion* and *prevention*. Promotion focus describes an individual's tendency to attend to opportunities to use eager strategies and achieve gains. Prevention focus describes an individual's tendency to engage in opportunities to use vigilant strategies and prevent losses. When the goals and affordances of a given task or situation align with the individual's regulatory focus, that individual is said to be in regulatory fit. Regulatory fit enhances the subjective value of activities, leading to increases in effortful engagement with those activities (Cooper et al., 2015). Regulatory fit can influence self‐reported measures of states such as motivation (e.g., Idson et al., 2004) and observable behavior in or performance on a task (e.g., Files et al., 2019; Worthy et al., 2007).

In the present study, we measured participants' prevention and promotion strengths with the Regulatory Focus Questionnaire (RFQ; Higgins et al., 2001) and randomly assigned them to carry out a speeded go/no‐go task with post‐trial feedback framed in terms of point gains, point losses, or an informative‐only control. In all conditions, the instructions emphasized the need to respond quickly on the *go* trials and to withhold responses on the *no‐go* trials. The 2‐point‐based feedback conditions assigned equal weight to speed and accuracy on the *go* trials and assigned much more weight to accuracy on *no‐go* trials. Regulatory fit theory would predict that participants with stronger prevention strengths would be more motivated and engaged by point loss‐based feedback, and those with stronger promotion strength would be more motivated and engaged by point gain‐based feedback. However, because response speed feedback was given on every *go* trial, response speed was expected to be at least somewhat motivationally relevant for all participants.

To preview the results, we found a strong effect of response time on CRN amplitude such that slower RTs resulted in more negative CRNs, and this effect was stronger for more promotion‐oriented people in the gain‐framed condition. We interpret these findings as consistent with the hypothesis that CRN reflects response time when it is motivationally relevant, and that this relationship also reflects subjective motivational relevance resulting from alignment between promotion orientation strength and a gain‐framed task (i.e., regulatory fit).

## 2 METHOD

### 2.1 Participants

Inclusion criteria were normal or corrected‐to‐normal visual acuity, normal color vision, and not having experienced neurological trauma. The voluntary, fully informed, written consent of participants in this research was obtained as required by Title 32, Part 219 of the CFR and Army Regulation 70‐25. All human subjects testing was approved by the Institutional Review Board of the US Army Research Laboratory under protocol 17‐166. Our target sample size was 90, which we selected to match the sample size in our previous work using a similar paradigm (Files, Pollard, et al., 2019). For that study, the sample size was expected to be sufficient to find a medium‐to‐large effect size in a 3 × 2 between‐subjects design based on an a priori sensitivity analysis. One hundred and twenty‐one participants (50 F and 67 M, four declined to answer), recruited via electronic message boards in Los Angeles, CA, met all inclusion criteria and completed the experiment. The mean age was 31.9 years (range 18–65). Data that were unusable due to experimenter error (10), technical problems (10), and excessive noise (3) were removed. An additional seven participant data sets were excluded because they had exceptionally high false alarm rates, suggesting inattention or a misunderstanding of instructions (a false alarm rate of 83% or higher on 10 or more blocks), leaving a total of 91 usable data sets.

### 2.2 Procedure

After giving informed consent, participants were screened for normal color vision (Ishihara 14‐plate test) and visual acuity (better than 20/40 in each eye with a Snellen 20' chart). Participants then took a set of questionnaires including the regulatory focus questionnaire. Past work has found the internal consistency (Cronbach's alpha) for the promotion scale and the prevention scale of the regulatory focus questionnaire to be .73 and .80, respectively, and the test‐retest reliability (Pearson product moment correlation) to be .79 and .81, respectively (Higgins et al., 2001).

The EEG cap and electrodes were applied, and then the participant was seated in a cool, dimly lit, acoustically isolated booth approximately 0.75 m from a computer monitor display. Three minutes of resting baseline data were collected, followed by providing instructions for the task. Participants were assigned to the gain‐framed, loss‐framed, or control feedback condition, and the instructions described how performance feedback would be displayed. Assignment to feedback condition was random, although after elimination of unusable datasets, the distribution was not equal across all conditions (26 gain, 35 loss, and 30 control). All analyses (see following sections) do not rely on equal variance or equal *n* assumptions. After the participant passed a two‐question comprehension check, the task began.

The task was a go/no‐go task using computer‐rendered images of human characters. The images were taken from preexisting simulated wartime environments used in past research and were meant to be ecologically valid for military contexts. We used a similar task in a previous study (Files, Pollard, et al., 2019), although the number and timing of trials differs here. The go stimulus was a computer‐rendered character holding a rifle. The no‐go stimulus was a similar character not holding a rifle. Stimuli were presented in randomized positions and at a random scale. Participants were instructed to respond as quickly as possible to the go stimulus by pressing a button on a response box and to withhold responses to the no‐go stimulus.

The task consisted of 20 blocks of 30 trials each (24 go and 6 no‐go). Each stimulus was visible for 0.4 s, and feedback appeared 1 s after stimulus onset. Feedback was visible for 0.5 s, and then a fixation cross appeared for a variable inter‐stimulus interval lasting between 1 and 2 s (uniform distribution). Stimuli were presented with a randomized scaling factor, so they were between 1° tall by 0.4° wide and 4.0° tall by 1.6° wide and at a location selected randomly from a rectangle centered on the display area that subtended 29° of visual angle by 2.1° of visual angle. On a randomly selected half of stimulus presentations, the image was mirror‐reversed.

The trial feedback depended on the type of trial and the response time. For the gain and loss conditions, number of points gained or lost were displayed in green if the response was correct or red if the response was incorrect. Go trials were worth up to 60 points with 30 of those being for responding before the deadline at 1 s and 30 for speed, such that faster responses gained more or lost fewer points. No‐go trials were worth 180 points. In the control condition, a green check was displayed with a response speed meter for correct responses and a red X was displayed for incorrect responses. On the right of the screen, a bar showed cumulative information about the block. In the gain condition, the bar began empty and filled up showing cumulative point gains. In the loss condition, the bar began full and emptied showing cumulative point losses. In the control condition, the bar filled after each trial regardless of response as a measure of progress through the block. After each block, summary feedback was presented breaking down speed and accuracy for go trials and accuracy for no‐go trials. Summary feedback was presented as points gained, points lost, or as percent accuracy and speed in ms in the gain, loss, and control conditions, respectively.

**FIGURE 1:**
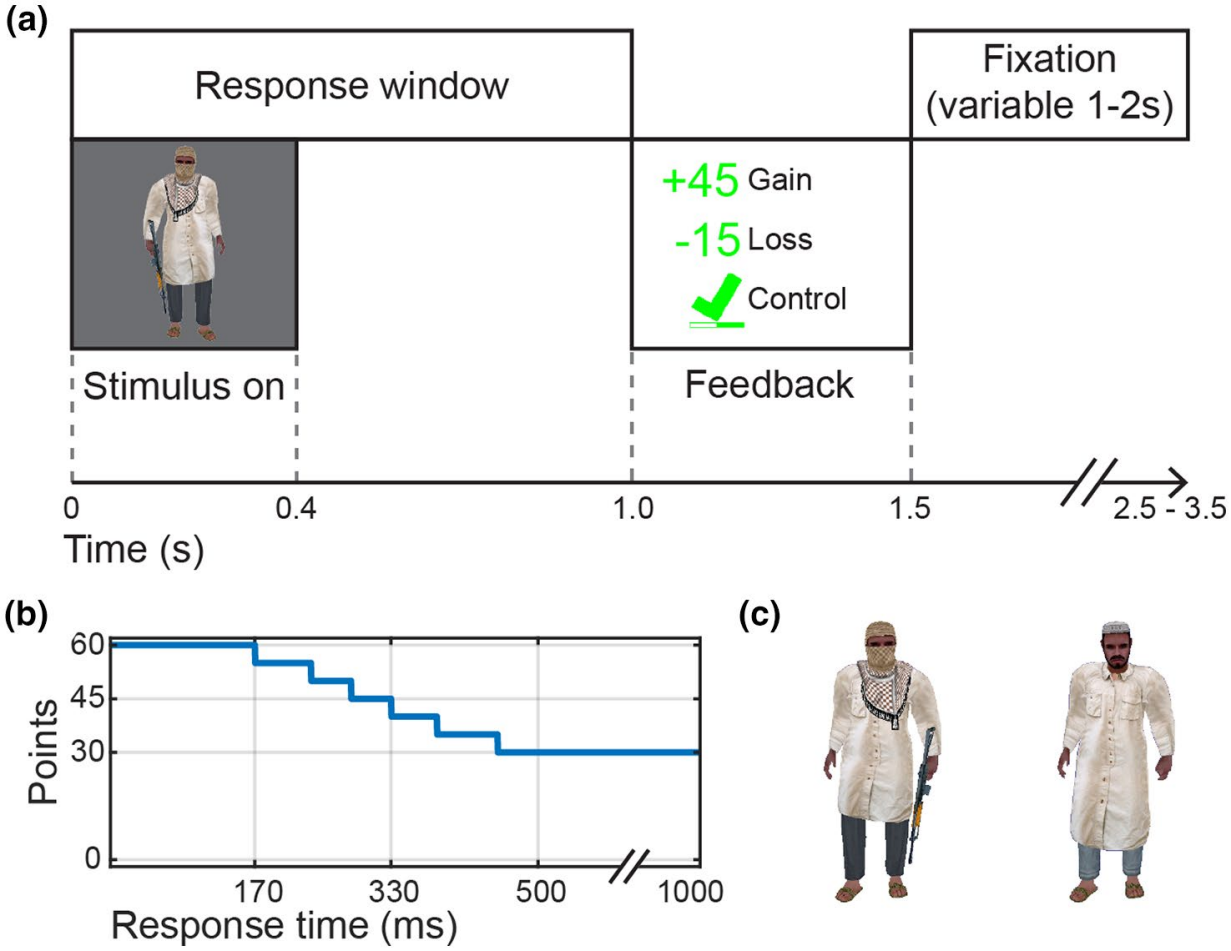
Method. The trial timeline (a) shows the timing of events within a single trial, including the fixation‐only period separating trials which randomly varied from 1–2 s (uniform distribution). The timeline also shows feedback as it appeared in the gain, loss, and control conditions. Participants could respond any time between stimulus onset and 1 s after onset. The response time points curve (b) shows that response speed was rewarded (or not penalized) for responses faster than 452 ms. The go stimulus and no‐go stimulus images (c) were rendered images of the same underlying 3D model in the same pose with different clothes and holding a rifle or not

Figure 1 shows the single‐trial timeline, the curve relating response time to number of points, and the stimulus images used in the experiment.

After the task, participants filled out the Intrinsic Motivation Inventory (Ryan, 1982) as a subjective motivation measure. Participants then completed a second task (described elsewhere) and an exit questionnaire. For full details of the procedure, including details of tasks and questionnaires not reported on here, please see https://osf.io/vzw4n/. Overall, the experiment took 2–3 hr.

### 2.3 EEG recording and reduction

EEG was recorded using a BioSemi Active 2 (input impedance 300 MOhm @ 50 Hz) setup with 64 active sintered Ag/AgCl electrodes placed in an electrode cap using a modified 10‐10 arrangement. Additional electrodes were placed on the mastoids, outside the external canthi of the eyes and above and below the left eye. To ensure adequate contact between electrodes and the scalp, DC offsets were required to be stable and no more than 30 µV from zero. Data were sampled at 2,048 Hz with an online 5th order cascaded integrator‐comb digital filter (−3 dB at 400 Hz) and were down‐sampled to 512 Hz for storage.

Raw data were high‐pass filtered at 0.2 Hz and band‐stop filtered at 60 Hz both using second‐order Butterworth filters. Noisy channels were identified by visual inspection and interpolated using neighboring electrodes, and then data were re‐referenced to the average. Independent components analysis (Bell & Sejnowski, 1995) with PCA reduction was used to remove components representing eye blinks and eye movements. Cleaned data were divided into non‐overlapping segments of 0.5 s. The FFT for each channel was computed, and channel pair spectral correlations were computed and averaged to characterize the cross‐channel spectral correlation for each segment. Segments with a cross‐channel spectral correlation more than 1.8 standard deviations from the mean over all segments were marked as bad. Following cleaning, data were low‐pass filtered at 30 Hz with a two‐pass sixth‐order Butterworth filter. In subsequent event‐related analyses, epochs overlapping bad segments were rejected from analysis.

A standard approach to computing ERN amplitude is to subtract response‐locked EEG for correct responses from response‐locked EEG for errors. This approach, resulting in the ΔERN, was considered inappropriate for the present experiment, because the response time distributions for false alarm responses were generally faster than the distributions of correct responses, and there was heterogeneity in response time distributions across participants. The ΔERN would not be free of stimulus‐evoked activity, because false alarms were more likely to occur during early visual responses to stimulus onset, but correct responses tended to occur later in visual processing of the stimulus onset and after stimulus offset. Moreover, the ΔERN can obscure effects that are due to changes in only the ERN or CRN (Meyer et al., 2017). Although the ΔERN has been shown to be more robust when its use is appropriate (Olvet & Hajcak, 2009a), here we extracted ERN and CRN separately.

CRN waveforms were extracted by first subtracting from each correct response trial the average over all of that participant's correct responses. This was meant to subtract out stimulus‐evoked potentials, leaving response‐related potentials and noise. We then extracted a response‐related epoch from 500 ms before that trial's button press to 500 ms post‐response. This resulted in a CRN waveform for each correct response. These CRN waveforms were baseline corrected over 500–400 ms pre‐response. ERN waveforms were extracted in the same way, but because false alarms were relatively rare, the stimulus‐evoked activity was based on the average of all no‐go trials. Past work has shown that as few as six error trials are sufficient for characterizing the ERN (Olvet & Hajcak, 2009a), but we carried out a subject‐level internal consistency analysis (Clayson et al., 2021) with data from participants with six or more usable trials to select participants with internal consistency greater than .8 (Clayson & Miller, 2017).

To characterize ERN and CRN amplitudes, we visualized the grand mean ERN and CRN waveforms at electrode FCz and selected windows around the respective negative peaks of plus and minus 50 ms. We used the average amplitude over that time window to characterize single‐trial ERN and CRN, rather than the peak amplitude within it, because the average over time is an unbiased estimate of component amplitude (Luck, 2005). Electrode FCz was selected based on previous work showing that the ERN is largest there (Gehring et al., 2011). Past work using different stimuli has shown that split‐half reliability for ERN is .84 and CRN is .98 using area measures on FCz, and test‐retest reliability is .70 for ERN and .82 for CRN with a 2‐week test interval (Olvet & Hajcak, 2009b) and with a 1.5–2.5 year test interval is .67 for ERN and .75 for CRN (Weinberg & Hajcak, 2011). Reliability analyses of the present data are included below.

### 2.4 Data analysis

Behavioral data were analyzed using Bayesian reduced‐rank multivariate regression (Files et al., 2019) implemented in Stan (Carpenter et al., 2017; Gelman et al., 2015). For the ERN and CRN data, there were several possible predictors and interactions of relevance, so we opted to include them all in a model with regularization to avoid overfitting. The regularization approach was to use regularized horseshoe priors (Piironen & Vehtari, 2017) on the regression coefficients, which shrinks most coefficients toward zero but allows some to be relatively large. A random intercept for each participant was used to partially account for individual variability, and Student's *t*‐distributed error terms limited the influence of extreme values. Specifically,

$$Y_{k}∼T\left(\nu ,B_{i}^{T}X_{k}+I_{j},\sigma_{g}\right),$$

$$B_{i}∼N\left(0,\Sigma_{i}\right),$$

$$I_{j}∼N\left(I_{0},\sigma_{I}\right),$$

$$\nu ∼G\left(2,1/10\right),$$

$$\sigma_{g}∼N^{+}\left(0,1\right),$$

$$\sigma_{I}∼N\left(0,1\right).$$

Here, *Y_k_* is either ERN or CRN for trial *k* from participant *j* in condition *i*. The symbols $T,N,N^{+}$, and G represent Student's *t*, normal, half‐normal and gamma distributions, respectively. Coefficients *B_i_* and predictors *X*_k_ are *P*‐vectors, where *p* = 11 is the number of predictors in the model. The predictors were response time, promotion strength, prevention strength, number of errors, and all two‐ and three‐way interaction terms not involving both promotion and prevention strengths. The standard deviation, $\Sigma_{i}$, of the prior on the regression coefficients *B_i_* was specified following (Piironen & Vehtari, 2017),

$$\Sigma_{i}=diag\left({\tilde{\lambda }}_{i,1}\tau ,{\tilde{\lambda }}_{i,2}\tau ,\ldots {\tilde{\lambda }}_{i,P}\tau \right)$$

$${\tilde{\lambda }}_{i}^{2}=\frac{c^{2}\lambda_{i}^{2}}{c^{2}+\lambda_{i}^{2}\tau^{2}},$$

$$c^{2}∼G^{‐1}\left(\frac{\nu_{0}}{2},\frac{\nu_{0}s^{2}}{2}\right),$$

$$\lambda_{i}∼C^{+}\left(0,1\right),$$

$$\tau ∼C^{+}\left(0,\tau_{0}\right),$$

$$\tau_{0}=\frac{p_{0}}{PC‐p_{0}}\frac{\sigma_{g}}{\sqrt{N}}.$$

Here, also, ${\tilde{\lambda }}_{i}$ and $\lambda_{i}$ are *P*‐vectors, *C* = 3 is the number of conditions in the experiment, *N* is the total number of trials. The expected number of non‐zero coefficients, $p_{0}$, was set to 9. The hyper‐parameters $\nu_{0}$ and *s* express the prior belief that coefficients that are not zero will follow a Student's *t* distribution with $\nu_{0}=20$ degrees of freedom and scale *s* = 1. The effect of these priors is to keep most coefficients close to zero but allowing some to be relatively large. ERN, CRN, and all predictors were *z*‐transformed prior to analysis. The symbol $C^{+}$ represents the half‐Cauchy distribution.

Models were fit using the RStan interface (Stan Development Team, 2018) to Stan version 2.18.0. We used eight independent chains with 8,000 warmup iterations and 1,000 post‐warmup iterations per chain. Chain mixing was assessed with $\hat{R}$, with the criterion that $\hat{R}$ for all parameters was less than 1.05. All parameters achieved an effective sampling ratio greater than 0.1.

For internal consistency and fixed‐effects dependability analysis, we fit the same model but with the addition of a mean‐zero random coefficient on response time, that is, $Y_{k}∼T\left(\nu ,B_{i}^{T}X_{k}+b_{j}rt_{k}+I_{j},\sigma_{g}\right)$ with the same prior on $b_{j}$ as on $B_{i}$. We computed subject‐level variance components and computed the internal consistency coefficient as a function of number of trials, $n_{j},$ from that subject and the error variance, $\sigma_{e,j}^{2},$ from that subject's trials:

$${\hat{Y}}_{j,k}=B_{i}^{T}X_{j,k}+b_{j}rt_{j,k}+I_{j}$$

$$\sigma_{m}^{2}=Var\left({\hat{Y}}_{k}\right)$$

$$\sigma_{e,j}^{2}=Var\left(Y_{j,k}‐{\hat{Y}}_{j,k}\right)$$

$$\phi_{j}=\frac{\sigma_{m}^{2}}{\sigma_{m}^{2}+\sigma_{e,j}^{2}/n_{j}}$$

We computed internal consistency for each participant and retained for modeling only those with internal consistency of .8 or greater.

Similarly, we computed variance components for group‐level estimates to identify the number of participants needed to obtain dependable fixed effects estimates as a function of number of subjects, *S*:

$${\hat{Y}}_{k}=B_{i}^{T}X_{k}+b_{j}rt_{k}+I_{j}$$

$$\sigma_{G}^{2}=Var\left(B_{i}^{T}X_{k}\right)$$

$$\sigma_{s}^{2}=Var\left(b_{j}rt_{k}+I_{j}\right)$$

$$\sigma_{e}^{2}=Var\left(Y‐\hat{Y}\right)$$

$$\phi_{G}\left(S\right)=\frac{\sigma_{G}^{2}}{\sigma_{G}^{2}+\frac{\sigma_{s}^{2}+\sigma_{e}^{2}/\hat{n}}{S}}$$

where $\hat{n}$ is the harmonic mean number of trials per subject and S is the number of participants.

Code and data to reproduce these analyses are publicly available at https://osf.io/7jbnd/.

## 3 RESULTS

**FIGURE 2:**
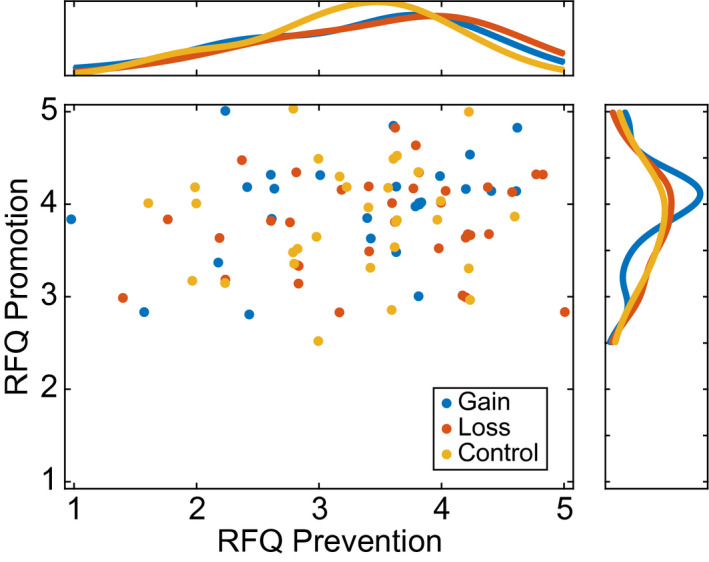
Regulatory Focus Questionnaire (RFQ) promotion and prevention subscale scores were weakly correlated (*r*(89) = 0.175, 95% CI [−0.047, 0.374]). Marginal panels show univariate distributions. Colors indicate feedback framing to which participants were randomly assigned. Data are jittered to reveal tied data points

Participant characteristics by experimental condition appear in Table 1. Additional details about the participants' characteristics are available online at https://osf.io/h8u2z/. Distributions of prevention and promotion strength are shown in Figure 2. Cronbach's alpha for RFQ promotion was .62, 95% bootstrapped CI [0.47, 0.73] using percentile bootstrap and 5,000 repetitions. Cronbach's alpha for RFQ prevention was .79, 95% bootstrapped CI [0.71, 0.85].

**TABLE 1:** Participant characteristics

Sample characteristics	Gain‐framed group		Loss‐framed group		Control feedback group	
	*N*		*N*		*N*	
Participants	26		35		30	
Sex	8F, 18M		20F, 15M		15F, 15M	
	*m*	*SD*	*m*	*SD*	*m*	*SD*
Age	28.1	11.7	31.2	11.2	32.6	14.6
Promotion strength	4.0	0.6	3.8	0.5	3.8	0.6
Prevention strength	3.3	0.9	3.5	0.9	3.2	0.8

**FIGURE 3:**
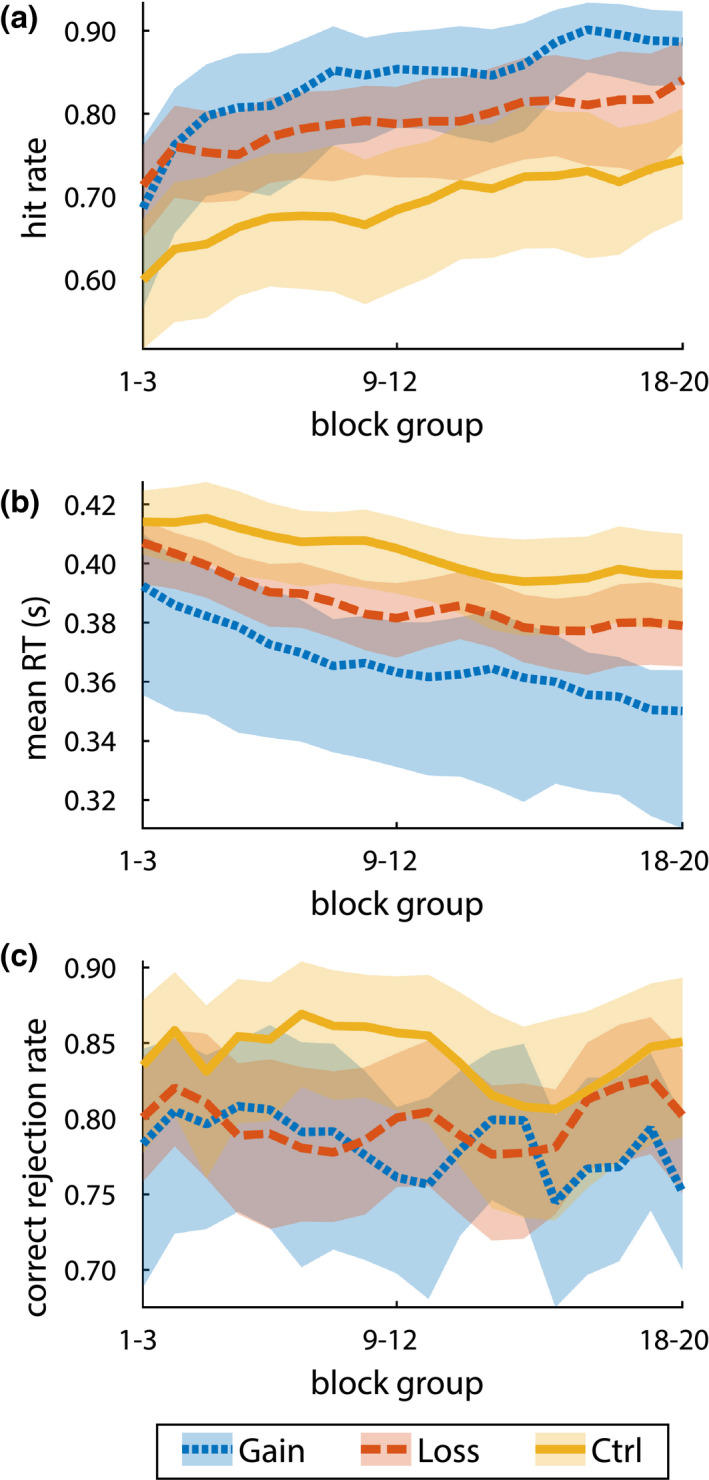
Hit rate (a) and mean response time (b) improved over the course of the task blocks, whereas correct rejection rate stayed approximately the same (c). Lines indicate group mean values pooled over three successive blocks, and shaded regions show bootstrapped 95% confidence regions. Colors indicate experimental condition

Behavioral performance generally improved over the course of the experiment (Figure 3). A reduced‐rank multivariate regression was run to see whether regulatory focus interacted with feedback framing to affect changes in task performance or self‐reported motivation. Initially, the model was fit with one latent dimension. Adding additional latent dimensions did not reliably improve the model fit as assessed with a Pareto‐smoothed importance sampling approximation of leave‐one‐out cross‐validation of expected log predictive density (Vehtari et al., 2019).

**FIGURE 4:**
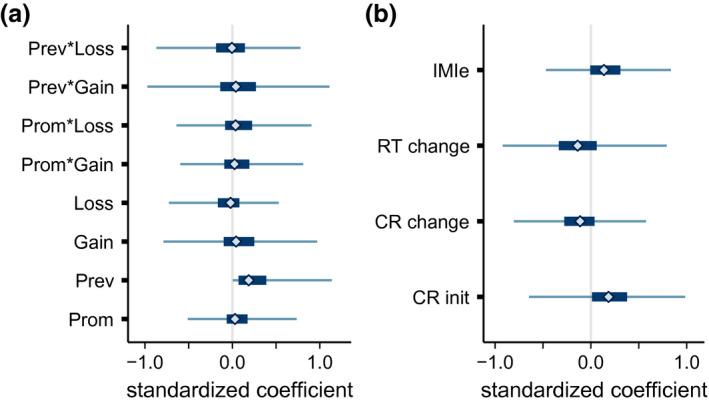
Posterior distribution summaries for (a) coefficients projecting predictors onto the latent performance variable and (b) coefficients projecting the latent performance variable onto the outcomes. Summaries show the central 95% (thin lines) 50% (thick lines) and mean (diamonds) of the posterior samples. Predictors were RFQ promotion, RFQ prevention, indicators for Gain‐framed condition and Loss‐framed condition, and their interactions. Outcomes were initial log odds‐transformed correct rejection (CR), change in log odds CR, change in response time (RT), and response to the intrinsic motivation inventory effort/enjoyment subscale (IMIe). The coefficient associated with prevention strength is constrained to be positive by the model

This analysis produced posterior distributions for the coefficients projecting the predictors onto a latent performance measure as well as for coefficients projecting that latent performance measure onto the observed performance measures (Figure 4). For reasons of identifiability, one of the coefficients in the model is fixed to be positive (here, the coefficient associated with RFQ prevention), but the posteriors for all other coefficients overlap with zero, so we cannot claim with confidence to know the sign of any relationships between regulatory focus and performance or self‐reported motivation.

Variance components, subject internal consistency, and fixed effects reliability estimates with posterior 95% credible intervals, along with additional details of the reliability analysis, are available in an online supplement, https://osf.io/wuzc2/ for CRN and https://osf.io/vsp3q/ for ERN. The internal consistency analysis of the CRN data found that four participants' data produced internal consistency measures lower than .8. For the ERN data, of the 77 subjects with six or more trials, 25 participants' data produced internal consistency measures lower than .8. The CRN fixed effects dependability analysis indicated dependability (mean and 95% credible interval) of .90 [.85, .93], .93 [.89, .95], and .92 [.88, .94] for gain, loss, and control groups, respectively. ERN fixed effects dependability was .56 [.21, .80], .68 [.31, .87] and .60 [.24, .83] for gain, loss, and control groups, respectively. All collected data are represented in group summaries, but the four participants with low internal consistency were excluded from statistical modeling of CRN data. Because of the low internal consistency of ERN among so many participants and the low fixed‐effects dependability of the ERN data, we do not present statistical modeling of the ERN data.

**FIGURE 5:**
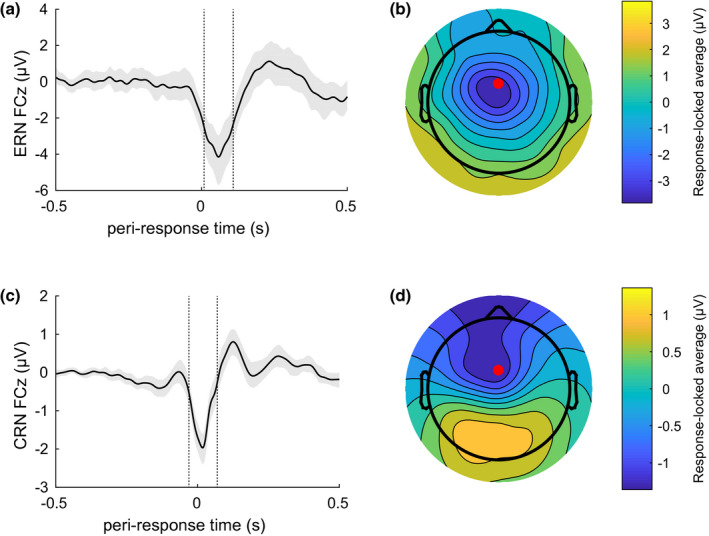
ERN grand average waveform (a) and scalp map (b) and CRN grand average waveform (c) and scalp map (d). Waveforms shown are from electrode FCz, indicated with a red dot on the scalp maps. Waveforms are aligned to the button press at time zero. Note the differences in scale between the four panels. Shaded regions in (a) and (c) are 99.9% bootstrapped confidence intervals (10k repetitions). Vertical dashed lines in the waveform plots indicate the time windows of 50 ms before and after their respective peaks used both for summarizing ERN and CRN amplitudes and for generating the scalp maps

Trial counts, waveform characteristics, and performance characteristics per group appear in Table 2. Grand average waveforms and scalp maps appear in Figure 5. The peak of the ERN waveform occurred at 59 ms after response. The grand mean ERN amplitude averaged from 9 to 109 ms relative to response was −5.90 μV with standard deviation 4.59 μV, computed from 77 subjects contributing mean 16.8, *SD* 0.8 trials; participants with fewer than six usable false alarm trials were not included. The peak of the CRN waveform occurred 20 ms post response. The grand mean CRN amplitude averaged from −30 to 70 ms was −1.17 μV with standard deviation 0.76 μV computed from 91 subjects contributing mean 257, *SD* 79.3 trials.

**TABLE 2:** ERP and performance summary

Performance characteristics	Gain‐framed group		Loss framed group		Control feedback group	
	*m*	*SD*	*m*	*SD*	*m*	*SD*
*Correct response negativity*						
Trials	258.54	93.32	257.5	81.32	255.00	65.47
ERP amplitude	−1.33	0.92	−1.18	0.66	−1.02	0.70
Hit response time (s)	0.40	0.07	0.43	0.05	0.46	0.61
Miss count	22.46	52.70	10.57	30.85	12.10	22.03
*Error response negativity*						
Trials	16.16	11.57	17.00	8.54	17.14	11.02
False alarm response time (s)	0.35	0.07	0.36	0.05	0.40	0.06
False alarm count	33.40	15.68	31.70	12.91	32.55	16.95

**FIGURE 6:**
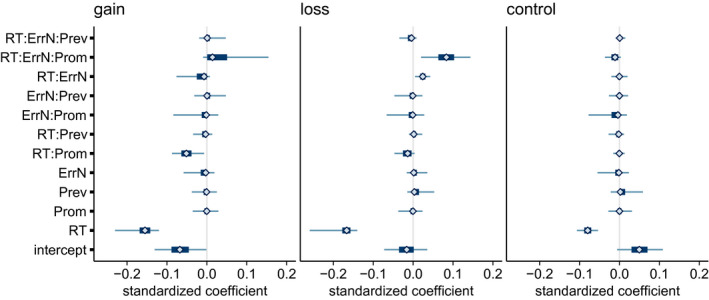
Posterior summaries of regression coefficient parameters relating standardized CRN amplitude to standardized response time (RT), RFQ promotion strength, RFQ prevention strength, number of miss errors (ErrN), and their interactions. Thin and thick lines show central 95% and 50% credible intervals. Diamonds show the median of the posterior sample

**FIGURE 7:**
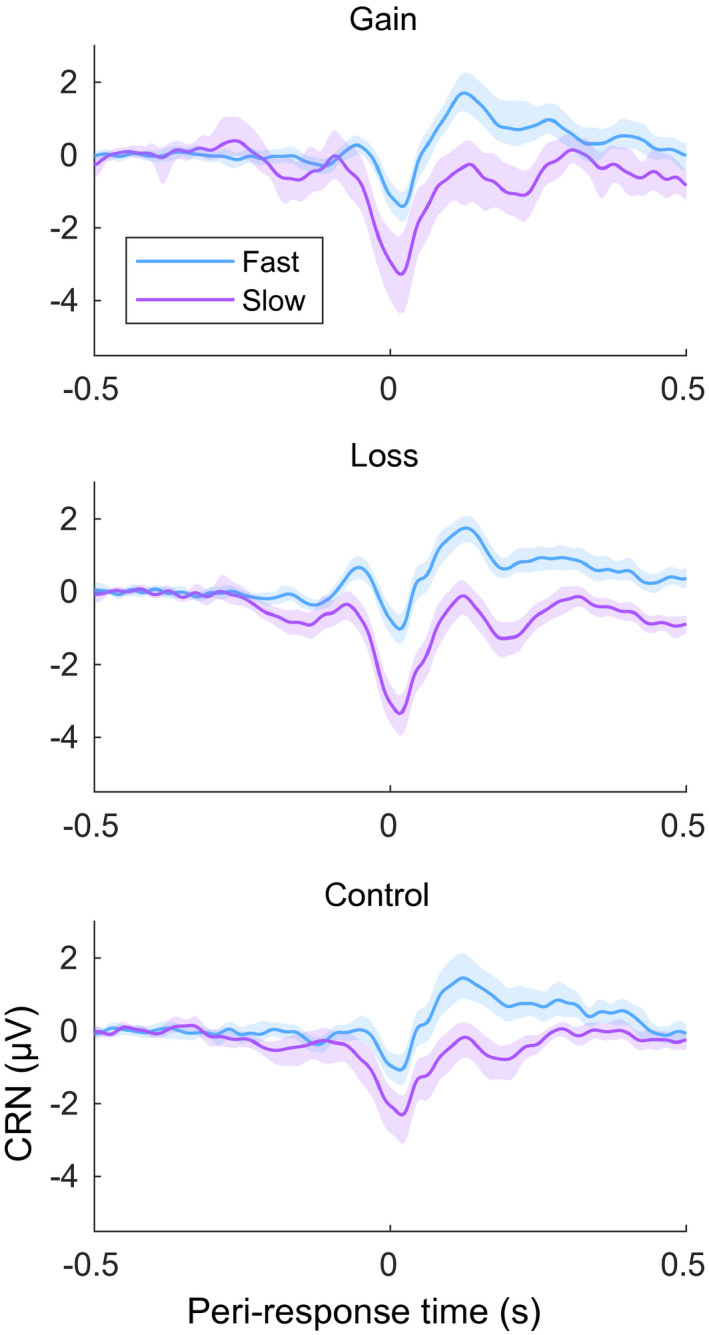
Correct response negativity waveforms on electrode FCz for the three feedback conditions. For visualization purposes, fast and slow response waveforms are separated by median split at 0.418 s. All waveforms are aligned to the button press at time zero. Shaded regions show bootstrapped 95% confidence regions (10k repetitions). Waveforms for Gain, *n* = 26, average 152.0 and 106.5 trials in fast and slow, respectively; Loss, *n* = 35, average 134.7 and 122.9 trials in fast and slow, respectively; and Control, *n* = 30, average 100.9 and 154.1 trials in fast and slow, respectively. In all conditions, slower responses are associated with a larger correct response negativity

**FIGURE 8:**
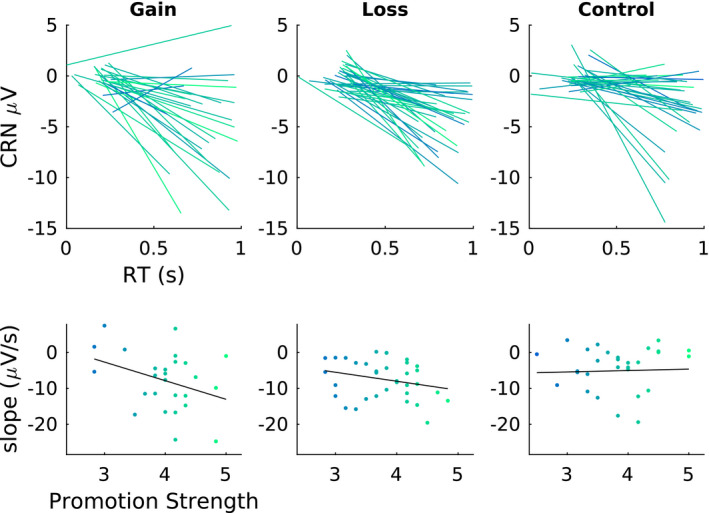
Graphical summary of relationship between correct response negativity (CRN), response time (RT), and regulatory focus promtion strength. Upper panels show lines of best fit between RT and CRN for each participant. These lines are fit with ordinary least squares without controlling for any other covariates. Line color indicates the indivdiual's promtion strength. Lower panels show the slopes of the lines in the upper panels against the corresponding participant's promotion strength score. Slopes were generally negative reflecting a more negative CRN as RT increased. The black line in the lower panel is the ordinary least squares line of best fit to the group data, illustrating the more negative slope associated with stronger promotion strength in the gain condition, less so in the loss condition, and absent in the control condition

Posterior summaries of the coefficient terms for the CRN model are in Figure 6. In all three conditions, the posterior distribution for the coefficient associated with response time was concentrated well below zero. The median and central 95% credible intervals were −0.15 [−0.23, −0.12], −0.17 [−0.26, −0.14] and −0.08 [−0.12, −0.05] for gain, loss, and control, respectively. These show that as response time increases, the CRN becomes more negative assuming an average promotion strength, prevention strength, and number of errors. CRN waveforms by response time and condition appear in Figure 7. Other coefficients also had posteriors concentrated far from zero. In the gain condition, the interaction of RT with promotion strength (−0.05, [−0.09, −0.01]) showed that the slope relating RT to CRN was steeper for individuals with stronger promotion strength and shallower for individuals with weaker promotion strength, given an average number of errors. A graphical summary of the relationship between RT, promotion strength, and feedback framing is in Figure 8. To further characterize the relationship between RT and CRN, we computed Pearson product moment correlations between response time and CRN for each participant, ignoring other predictors. In the gain condition, median correlation was *r* = −.15, 25th and 75th percentiles [−.22, −.04], in the loss condition it was *r* = −.15 [−.22, −.07], and in the control condition it was *r* = −.06 [−.16, .00]. Moreover, the posterior of the three‐way interaction between RT, promotion strength, and error number was concentrated in the positive direction, although the 95% credible interval included zero (0.01, [−0.01, 0.15]), suggesting that the promotion‐associated‐steepening of the slope relating RT to CRN might be exaggerated in individuals who committed fewer errors and moderated in those who committed more.

The interaction of RT with RFQ promotion was smaller in the loss than the gain condition and the central 95% credible interval included zero (−0.01, [−0.05, 0.00]). The three‐way interaction of RT, number of errors, and RFQ promotion strength in the loss condition was somewhat larger compared with that in the gain condition (0.08, [0.02, 0.14]). Unlike in the gain condition, in the loss condition, there was a positive interaction of RT with error number (0.02, [0.00, 0.04]), such that the slope of the line relating RT to CRN amplitude was shallower in those who committed more errors and steeper in those who committed fewer. In the control condition, the three‐way interaction of RT, error number, and RFQ promotion was negative (−0.01, [−0.04, 0.00]), in contrast to the largely positive corresponding effects in the gain and loss conditions.

## 4 DISCUSSION

The hypothesis that the CRN reflects the value of a response was supported. In all conditions, more negative CRNs were associated with longer RTs. This is consistent with the idea that the CRN tracks the subjective value of a response, as participants were instructed to respond as quickly as possible, feedback after each *go* trial indicated the speed of the response, and we directly rewarded speed. In the gain and loss conditions, speed was explicitly rewarded (tied to points), whereas in the control condition, speed was not associated with points. The point‐based conditions established a task in which responding slowly was functionally equivalent to commission of a minor error, even when an accurate response was given. The effect of RT on CRN amplitude was considerably smaller in the control condition compared with the gain and loss conditions, as would be expected if reward‐induced motivation led the participants to more strongly perceive slowness as a minor form of error.

Our findings could also be consistent with a mechanism based on partial‐error effects. If a participant were to initiate an erroneous motor response, correct it, and then ultimately respond accurately (Masaki & Segalowitz, 2004; Matsuhashi et al., 2021), this could result in a relationship between slower RT and larger amplitude CRN. Such a scenario would be consistent with the hypothesis that CRNs represent a negative subjective valuation of suboptimal responding. A subtle difference is that the suboptimal responding discussed by Masaki and Segalowitz (2004) and Matsuhashi et al. (2021) takes the form of a partial error, as measured by a motor response initially directed toward an incorrect button press or button release. The experience of the partial error leads to a CRN, whereas the correction of this partial error slows RT. The relationship between CRN amplitude and RT may thus be due to “farther along” motor errors both being experienced as more erroneous and also taking longer to rectify.

However, this is unlikely to be the best explanation for our findings. In our study design, there was no incorrect button to press on go trials. Participants had to withhold button presses (not move) on the no‐go trials. Any partial errors (inappropriate motor initiations) could thus only be experienced on the no‐go trials and thus cannot explain the CRN versus RT relationship we found in our go trials. It is possible that an erroneous non‐motor process, perhaps perceptual or inhibitory, that occurs before a successful button press might be perceived by the participant as a partial error and might also take extra time to resolve, a situation analogous to the motor findings. Disentangling this mechanism from the perception of slowness itself as a form of error would require additional focused research, perhaps involving the inclusion of no‐feedback conditions, eye‐tracking, and muscle sensors.

The observation that more negative CRNs were associated with longer RTs is also consistent with the hypothesis that CRN is a marker of attentional control or heightened vigilance on certain trials (Matsuhashi et al., 2021; van Noordt et al., 2015, 2016). Under this hypothesis, trials on which a participant focused more attention, for whatever reason, are likely to have larger amplitude CRN (van Noordt et al., 2015, 2016) and to involve slower RT; a participant need not subjectively experience suboptimal responding as an error for this to occur. However, in our paradigm, the only reason or motivation that was systematically varied was the degree to which optimal responding was subjectively rewarded. We varied the subjective reward value of optimal responding by explicitly rewarding speed in some conditions and by placing participants in different degrees of regulatory fit. Without a motivation component, the attentional control hypothesis alone would not predict that points‐based feedback or regulatory fit would affect the RT versus CRN relationship. These interventions explicitly change motivation parameters and the subjective value of response speed. If changes in attentional control occurred in our participants, these changes likely occurred alongside, or in response to, motivational and subjective factors. It is possible that attention is a mediating factor through which motivational or subjective value differences influence RT and/or CRN. Additionally, if attention and motivation are non‐mutually‐exclusive phenomena (i.e., different levels of analysis), neurologically or psychologically, then these hypotheses are not in conflict. Future research can work to disentangle these elements or establish mediating effects.

The effects of regulatory focus we observed were small and at the edge of the sensitivity afforded by our data, as evidenced by the proximity of the credible interval to zero, but we interpret them as providing converging evidence for the relevance of motivation to the RT versus CRN relationship. The general regulatory fit hypothesis that alignment between regulatory focus and situational factors—here, feedback framing—should lead to increased subjective value was partially supported. Specifically, stronger promotion focus was related to a more extreme relationship between RT and CRN in the gain‐framed feedback. This result is consistent with stronger promotion orientation leading to a relative increase in the subjective value of fast responses and a corresponding decrease in the subjective value of slow responses. A similar but less pronounced pattern arose in the loss feedback condition and was absent in the control condition.

Past work has shown that reward‐related activity in the ventral striatum of the basal ganglia is related to promotion orientation strength in a gains‐oriented task, such that stronger promotion orientation is associated with less reward‐related activity (Scult et al., 2017). The authors offered two possible explanations for this result: if people with a stronger promotion orientation experience smaller subjective reward, it could motivate those people to actively seek and attend to opportunities for larger rewards. Another possibility is that the relationship between objective gains and subjective reward is exaggerated with stronger promotion orientation. This could lead a person with relatively strong promotion orientation to experience larger subjective reward from gains and to experience smaller subjective reward from non‐gains relative to a person with weaker promotion orientation (Scult et al., 2017).

In our study, the main effect of promotion strength on CRN amplitudes was concentrated around zero, so it does not seem that the sensitivity to point gains or losses is generally increased or decreased with promotion strength. However, the interaction of promotion strength with RT suggests that promotion strength is associated with an increased sensitivity to rewards that are larger or smaller than average, consistent with an exaggerated relationship between gains and reward‐related neural activity (Scult et al., 2017). That the interaction of promotion strength with response time was strongest in the gain‐framed feedback condition is consistent with the predictions from the general regulatory fit hypothesis that alignment between regulatory focus and situational factors—here, feedback framing—should lead to increased subjective value. However, other results predicted by the general regulatory fit hypothesis were not obtained. No behavioral effects of regulatory fit were observed, and there were no effects of prevention focus on the CRN or the loss‐framed condition. The absence of effects of prevention strength in the CRN is also consistent with the lack of effect of prevention strength on reward‐related activity in the basal ganglia (Scult et al., 2017).

In the loss‐framed condition, there was a small positive coefficient on the interaction between RT and number of errors, such that the overall relationship between RT and CRN was flattened in those participants who committed more than the average number of errors and was exaggerated in those who committed fewer. The relationship between RT and CRN amplitude also interacted with the number of errors in the loss‐framed feedback condition (and to a lesser extent in the gain‐framed condition): the interaction of RT, number of errors, and promotion orientation was associated with a positive coefficient on the CRN. This means that for individuals with an above‐average promotion strength and/or an above‐average number of misses, slower RTs were associated with less negative CRNs (i.e., smaller subjective value). These interactions are complicated, but motivational factors might offer a tentative explanation. The task instructions and structure created a trade‐off between speed in the *go* trials and accuracy on the *no‐go* trials. Some participants might have been more motivated to try to find a balance that yielded fewer false alarm errors but slower correct responses, whereas others might have tended toward faster responses. Regulatory fit theory would predict that people with a stronger promotion orientation in the loss‐framed condition might have found the feedback non‐motivating or irrelevant and disengaged from the task.

False alarm errors were relatively rare, so there was insufficient data to reliably model the relationship between ERN, regulatory focus, and feedback condition. In our experiment, participants with as many as 18 usable ERN trials were found to not meet our internal reliability criterion. This contrasts with past work obtaining reliable ERN estimates from as few as six trials (Olvet & Hajcak, 2009b), highlighting the importance of evaluating reliability on a per‐experiment basis (Clayson, 2020).

Although possibly due to the low number of ERN trials, the topographies of the ERN and CRN we observed were not very similar, possibly suggesting different sets of neural generators. These two patterns bear a striking similarity to those reported by Endrass and colleagues (2012; see their Figure 4), who support a two‐process model of response‐related activity. In that account, two patterns of neural generators contribute differentially to the CRN and the ERN, with one pattern possibly reflecting response correctness and task difficulty, and the other reflecting outcome‐independent response monitoring. The current study was not aimed at disentangling these two possible generators or their functional significance, but the possibly distinct topographies of the CRN and ERN are consistent with the hypothesis that the CRN and ERN have distinct neural generators.

### 4.1 Practical implications

Designers typically add game‐like elements to an experience (sometimes referred to as gamification) with the intention of increasing the motivational relevance of that experience for its users (Nacke & Deterding, 2017; Sailer et al., 2017). The success of this approach in improving objective outcome measures is mixed; in a meta‐analysis, 61% of studies showed a positive average effect of gamification (Seaborn & Fels, 2015). One possible explanation for the inconsistent success of gamification is that it takes a one‐size‐fits‐all approach (Files, Pollard, et al., 2019; Hanus & Fox, 2015), but different people respond differently to the addition of different game elements.

In past work with a similar paradigm, we showed that regulatory focus prevention strength was associated with differential performance under gain, loss, or no‐points feedback (Files, Pollard, et al., 2019). Here, we show that a neural correlate of subjective motivation was associated with regulatory focus promotion strength also in a feedback‐dependent relationship. However, we observed no strong effects on performance of regulatory focus or feedback framing in the present study. This behavioral difference is likely due to differences in the task structure between the two experiments. Both used a go/no‐go structure, but in the present study, the response deadline was relaxed (1 s vs. 0.5 s), stimulus onset time was jittered, and there were fewer trials. These differences led to the experiments affording different strategies, and task strategic affordances are relevant for regulatory fit (Dijk & Kluger, 2011; Spiegel et al., 2004).

So although we show support for the hypothesis that individuals differ in their responses to point‐based feedback, the relationship between feedback framing, regulatory focus, motivation, and task performance is complex. More work is needed before generalizable design principles for individualized gamification can be offered. However, we see regulatory focus as an easy to measure, promising possible predictor of an individual's response to the addition of point‐based game elements.

Another possible practical implication for the present findings would be to use the CRN amplitude as an implicit reporter of subjective motivation in the context of a brain‐computer interface. We asked participants to rate their subjective motivation, and we saw no effect of regulatory focus or feedback framing on reported motivation. This could suggest that the CRN effects do not actually reflect subjective motivation, or it could suggest that after‐the‐fact subjective reports might not be a very sensitive measure (Fulmer & Frijters, 2009). Either way, the effects on CRN amplitude were robust but small in magnitude. As such, we do not see CRN amplitude as a promising implicit reporter of subjective motivation in practical contexts. However, future work might sharpen or enhance this effect, yielding a more practically useful signal. CRN might be an implicit reporter of attention (Matsuhashi et al., 2021; van Noordt et al., 2015, 2016) more broadly. Future studies could look at relationship between CRN values and various measures of attention.

### 4.2 Limitations

In addition to the low number of false alarm trials, there are some other aspects of this study that could limit the generalizability of the findings. The feedback manipulation we used was between‐subjects, so we cannot definitively rule out group differences arising due to sampling error. Our smallest group size was 24, which is somewhat larger than the average group size reported in a recent survey of methodology in event‐related potentials studies (Clayson et al., 2019), but it is not so large as to eliminate concerns about sampling error. Indeed, the participants assigned to the gain condition were slightly younger and included more males compared with the other groups. The relationships between regulatory focus, number of errors, RT, and CRN are fairly small effects, and they differed by condition. However, the relationship between CRN and RT was found in all groups and was of comparable size in both the points‐based feedback groups, which might partially assuage concerns about the smallish sample size per group on the reliability of that particular finding.

The stimuli we used were naturalistic, rather than abstract. This could potentially limit the generalizability of our findings to other kinds of stimuli. The post‐task questionnaire asked participants to indicate, via free‐text response, how they felt about the activity and what they found exciting or agitating about it. Most participant responses were short and indicated positive or negative emotions when succeeding or failing respectively during the go/no‐go task. One response indicated strong emotions resulting from the stimuli used: “when I wrongly clicked on the civilian, I felt I was shooting innocent people.” This is intriguing, because we did not describe the study activity to participants as a shooting task but rather as identification of possible threat versus non‐threat stimuli. With the current data, we are unable to address whether participants' emotional response influenced task performance or the associated response‐evoked potentials. We note, however, that several practical applications of the go/no‐go task involve stimuli with strong emotional associations (Biggs et al., 2015; Hamilton et al., 2019; Houben & Jansen, 2011; Houben et al., 2011) in order to achieve ecological validity.

### 4.3 Conclusion

Here, we demonstrated a strong association between response time and CRN amplitude, and this effect was larger when response time was explicitly rewarded (i.e., associated with points‐based rather than with informative feedback). We suggest that the RT/CRN relationship depends on response time being motivationally relevant, with large amplitude CRNs representing a subjective reaction to commission of what may be perceived as a miniature error—a slower‐than‐desired response. The observation that an increase in regulatory focus promotion strength is associated with a steeper RT/CRN relationship in the points‐gain condition possibly reflects an exaggerated subjective valuation of points for individuals with stronger promotion focus in a state of regulatory fit.

## AUTHOR CONTRIBUTIONS

**Benjamin Taylor Files:** Conceptualization; Formal analysis; Investigation; Methodology; Visualization; Writing‐original draft. **Kimberly Anne Pollard:** Conceptualization; Methodology; Writing‐review & editing. **Ashley Haya Oiknine:** Data curation; Investigation; Methodology; Writing‐review & editing. **Peter Khooshabeh:** Conceptualization; Methodology; Supervision; Writing‐review & editing. **Antony Damian Passaro:** Conceptualization; Data curation; Investigation; Methodology; Project administration; Software; Supervision; Validation; Visualization; Writing‐review & editing.
